# Impact of a Nomadic Pastoral Lifestyle on the Gut Microbiome in the Fulani Living in Nigeria

**DOI:** 10.3389/fmicb.2019.02138

**Published:** 2019-09-13

**Authors:** Ayorinde O. Afolayan, Funmilola A. Ayeni, Christine Moissl-Eichinger, Gregor Gorkiewicz, Bettina Halwachs, Christoph Högenauer

**Affiliations:** ^1^Department of Pharmaceutical Microbiology, Faculty of Pharmacy, University of Ibadan, Ibadan, Nigeria; ^2^Division of Infectious Diseases and Tropical Medicine, Department of Internal Medicine, Medical University of Graz, Graz, Austria; ^3^BioTechMed, Interuniversity Cooperation, Graz, Austria; ^4^Institute of Pathology, Medical University of Graz, Graz, Austria; ^5^Theodor Escherich Laboratory for Microbiome Research, Graz, Austria; ^6^Division of Gastroenterology and Hepatology, Department of Internal Medicine, Medical University of Graz, Graz, Austria

**Keywords:** gut microbiota, Fulani, Jarawa, lifestyle, diet

## Abstract

The co-evolution of the gut microbiota with its human host has revolutionized our current scientific viewpoint about the contribution of diet and lifestyle on human health. Most studies so far have focused on populations living in the United States and Europe or compared those with communities from other geographic areas in the world. In order to determine the taxonomic and predicted functional profile of the gut microbiome of a hitherto unstudied human community, we investigated the phylogenetic diversity of the gut microbiota in a community of Fulani nomadic pastoralists, and their semi-urbanized neighbors – the Jarawa. The Jarawa reside in a city (Jos) in the north-central part of Nigeria, and are adapted in part to a westernized lifestyle. The nomadic Fulani lifestyle resembles a mix of Paleolithic and Neolithic lifestyle patterns with a greater predisposition to diseases. The fecal microbiota of the Fulani and the Jarawa were characterized by paired-end Illumina MiSeq sequencing of the 16S rRNA gene, followed by downstream bioinformatics analysis of the sequence reads. The Fulani harbored increased numbers of signatures of microbes that are known to be associated with a foraging lifestyle such as the Bacteroidetes, Spirochaetes, and Prevotellaceae, while the Jarawa were dominated by signatures of Firmicutes, Ruminococcaceae, Lachnospiraceae, and Christensenellaceae. Notably, the gut microbiota of the Fulani showed less taxonomic diversity than those of the Jarawa. Although they reside in the same geographical zone, microbial community composition was significantly different between the two groups. Pathogens were predicted to be more abundant in the gut microbiota of the Fulani than of the Jarawa. Predicted pathogenic pathways and pathways associated with the breakdown of fiber-rich diet were enriched in the Fulani, including glutathione metabolism, while pathways associated with the consumption of low-fiber diet and xenobiotics, including fructose and mannose metabolic pathways, and nitrotoluene degradation pathways, respectively, were enriched in the Jarawa. Significant differences in composition between both groups were likely due to differences in diet and lifestyle and exposure to pathogens. These results suggest that microbial diversity may not always be higher in non-industrialized societies than in westernized societies, as previously assumed.

## Introduction

**FIGURE 1:**
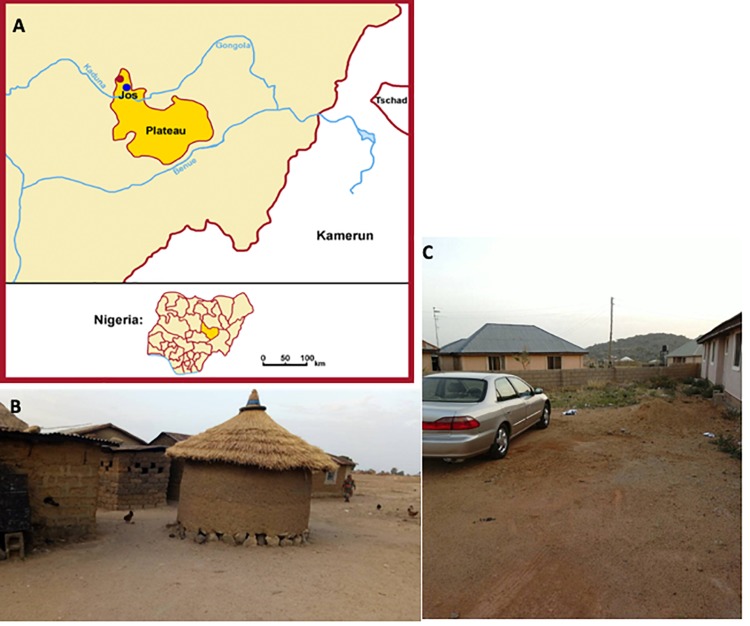
Location, scenery, and lifestyle patterns of nomadic Fulani and Jarawa (non-Fulani). The pictures show the geographical location of the Fulani (red dot) and the Jarawa (non-Fulani, blue dot) studied **(A)**, a typical nomadic Fulani house **(B)**, and a typical Jarawa (Non-Fulani) scenery of the area studied **(C)**.

The gastrointestinal tract of humans is home to an abundant and diverse consortium of microorganisms collectively known as the gut microbiota (Martínez et al., 2015). They are known to impact metabolism, physiology, and health of the host (Kau et al., 2011; Ramakrishna, 2013). Studying the variations of gut microbiota across human populations on various continents suggests that human gut microbiota is influenced by environmental adaptation. Comparative studies have shown that there is an integral relationship between lifestyle factors and the composition of the gut microbial community (Yatsunenko et al., 2012; Schnorr et al., 2014; Martínez et al., 2015). Most studies, however, have focused on Americans and Europeans, while only a limited number of studies have investigated populations on other continents with distinct differences in their mode of life (Gomez et al., 2016; Gupta et al., 2017; Ayeni et al., 2018). Groups selected for this study were from two areas of the Plateau State (the city of Jos, and a rural area within the town of Jengre), both in North-Central Nigeria (Figures 1A–C). Nigeria in West Africa is the most populous country in Africa and the 7th most populous country in the world. The World Factbook published by the Central Intelligence Agency (2016) shows that there are two major ethnic groups in the North-Central zone of Nigeria, namely the Fulani and the Hausa, who make up 29% of the Nigerian population. The Jarawa are one of the tribes that speak the Hausa language, and they (along with other Hausa-speaking tribes) are often erroneously referred to as Hausa. The majority of the Fulani in Nigeria are agro-pastoralists with a pastoral nomadic lifestyle. Although there is some interaction with the Hausa and other ethnic groups, their traditional lifestyle remains distinctly intact. The Fulani only mingle with other ethnic groups to sell their cattle or engage in other trade. They hardly ever intermarry, and they do not permit undue access to their community without permission from the head of the community. The nomadic Fulani commonly migrate seasonally in search of richer pasture for their cows. The Fulani reside not only in Nigeria but also in large numbers in Ghana, Guinea, Burkina Faso, Niger, Chad, Mali, Sudan, and Cameroon. The Fulani consume fiber-based foods (Lockett et al., 2009). The Jarawa eat meat regularly and consume a low-fiber diet with processed foods (pasta, spaghetti, refined rice, and other fast foods) along with most of the food (high-fiber diet) and fermented drinks also consumed by the Fulani. Traditional lifestyle, which correlates with consumption of crude, high-fiber diets rather than low-fiber, highly processed foods, as in a westernized diet, has a major impact on the composition and metabolic function of the intestinal microbiota and is considered to be negatively associated with predisposition to metabolic diseases and obesity (Turnbaugh et al., 2009; De Filippo et al., 2010). The effect of diet and lifestyle on the gut microbiome has generated considerable interest because these may have an impact on human health and are considered as a possible mechanism driving diseases of civilization (Ramadass et al., 2017).

We performed the study since we assumed that the very unique primal nomadic lifestyle of the Fulani has a major impact on the composition and the functional characteristics of the gut microbiota. For comparison we chose the urban Jarawa population, living in the same geographic region but with a more westernized lifestyle, including consumption of industrial processed food.

## Results

### Differences Between Profiles of Bacterial Communities Derived From Fecal Samples of the Fulani and the Jarawa

**FIGURE 2:**
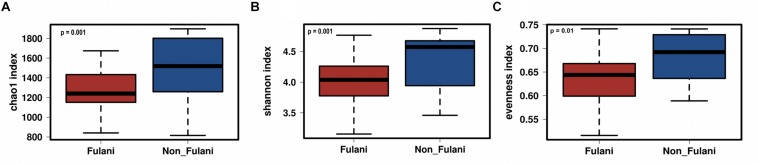
Differences in parameters of taxonomic alpha-diversity between Fulani and Jarawa (non-Fulani). Boxplots show a significantly lower Chao1 index of microbial richness **(A)**, a significantly lower Shannon index of microbial diversity **(B)**, and significantly lower evenness index **(C)** in the fecal microbiota of Fulani (red boxes), compared to Jarawa (non-Fulani, blue boxes). Mann–Whitney *U*-test was used with FDR-corrected *p*-values of **(A)** 0.001, **(B)** 0.001, and **(C)** 0.01.

**FIGURE 3:**
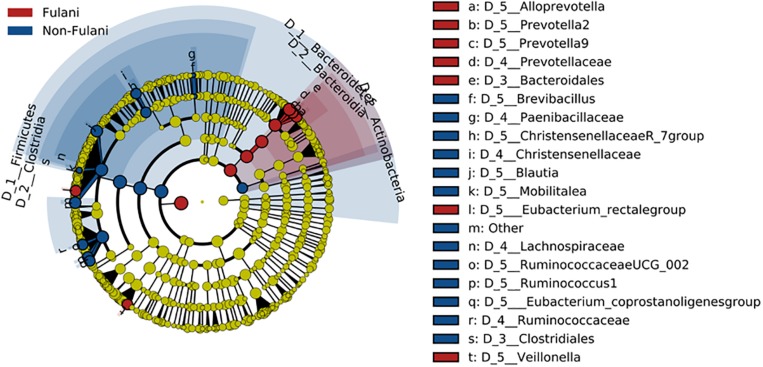
Cladogram of LEfSe analysis showing different abundance of microbial taxa in the gut of the Fulani (red) and the Jarawa (non-Fulani, blue).

**FIGURE 4:**
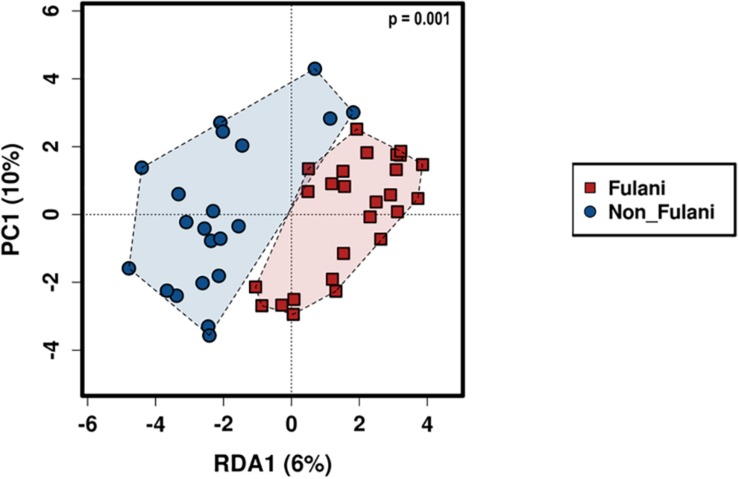
Beta-diversity. RDA plot based on Bray–Curtis distances shows patterns of significant separation between the Fulani (red boxes) and the Jarawa (non-Fulani, blue circles) at the OTU level (*p* = 0.001). The Calypso online software (http://cgenome.net:8080/calypso-8.84/faces/uploadFiles. xhtml) was used to perform RDA on log-transformed data. *P*-values were calculated using 999 Monte Carlo permutations and were corrected for multiple testing using Bonferroni correction.

Fecal samples were obtained from 28 healthy Fulani (13 males and 15 females, mean age = 19 years, *SD* = 16) and 22 healthy Jarawa (non-Fulani, 9 males and 13 females, mean age = 29 years, *SD* = 18). DNA extraction was performed and the hypervariable region 4 (V4) region of the microbial 16S rRNA gene was sequenced using the Illumina MiSeq platform, resulting in 1,265,159 high-quality paired-end reads with an average of 25,303 ± 5,596 reads per subject. Reads were clustered into 9,635 operational taxonomic units (OTUs) at 97% identity. Compared with the Jarawa, the Chao1 and the Shannon diversity indices revealed a significantly lower alpha diversity and evenness in the Fulani (Figures 2A–C). In total, 10 bacterial phyla were detected, with Firmicutes (Fulani vs. non-Fulani: 66.38 vs. 77.63%, *p*-value = 0.047), Bacteroidetes (Fulani vs. non-Fulani: 23.23 vs. 13.8%, *p*-value = 0.04), Proteobacteria (Fulani vs. non-Fulani: 8.49 vs. 5.50%, not significant), and Actinobacteria (Fulani vs. non-Fulani: 0.54 vs. 1.27%, *p*-value = 0.04) as the most dominant phyla in both ethnic groups (Table 1). The highest percentages of reads were assigned to the genus *Prevotella* 9 (Fulani vs. non-Fulani: 16.58 vs. 10.22%, *p*-value = 0.039), *Clostridium sensu stricto* 1 (Fulani vs. non-Fulani: 11.69 vs. 6.34%, not significant), *Blautia* (Fulani vs. non-Fulani: 2.96 vs. 6.64%, *p*-value = 0.045), and *Faecalibacterium* (Fulani vs. non-Fulani: 4.94 vs. 3.78%, not significant). The Fulani showed a trend toward a lower Firmicutes/Bacteroidetes ratio than the Jarawa (Fulani vs. Jarawa; 2.9 vs. 7.3%, *p* = 0.08997, Pearson’s Chi-squared test). Linear discriminant analysis effect size (LEfSE) was performed on different taxonomic levels (Supplementary Table 7). The results revealed several significantly discriminant microbial taxa (*p* < 0.05, LDA > 2) when Fulani and Jarawa sequence data were compared, see Figure 3, Table 1, and Supplementary Figure 2. The Fulani had significantly higher levels of Bacteroidetes and significantly lower levels of Firmicutes than the Jarawa (Figure 3, Table 1, and Supplementary Figures 1, 2). Signatures of the genera belonging to the phylum Bacteroidetes (*Prevotella* and *Alloprevotella*) were significantly higher in the Fulani than the Jarawa, while signatures of the genera belonging to the phylum Firmicutes (*Blautia*, *Ruminococcus* 1, Ruminococcaceae UCG002, and Christensenellaceae R7 group) were significantly higher in the Jarawa than in the Fulani (Figure 3). The Jarawa were enriched in signatures belonging to the archaea domain (Figure 3 and Table 1). Members of the order Lactobacillales (Fulani vs. non-Fulani: 0.2 ± 1.25% vs. 0.4 ± 0.6%; *p* = 0.44 including *Lactobacillus*, *Weissella*, *Lactococcus*, and *Leuconostoc*) were not significantly different between the study groups, although the Fulani are known to consume fermented raw and cooked milk regularly. Although Spirochaetes at phylum level was significantly enriched in the Fulani (Table 1), there was no significant difference in the relative abundance of *Treponema* signature (Fulani vs. non-Fulani: 0.3 ± 0.42% vs. 0.1 ± 0.1%; *p* = 0.072). When the gut microbial communities of the Fulani and the Jarawa were compared at the OTU level using the RDA plots based on Bray–Curtis distances, distinctness between both groups was observed (Figure 4 and Supplementary Figure 4). ADONIS analysis revealed that the differences in the gut microbial community composition between the Fulani and the Jarawa were statistically significant at genus and OTU level (*p* = 0.00333 and *p* = 0.000666, respectively). ANOSIM of Bray–Curtis distances revealed clustering of samples based on the groups (Fulani vs. Jarawa), at genus and OTU levels (Genus: *R* = 15.9%, *p* = 0.002; OTU: *R* = 22%, *p* = 0.001). As age is a factor influencing the composition of the intestinal microbiome, we also tested for differences between children and adults/adolescents that might have impacted the findings between the two groups, but found no significant differences in alpha and beta diversity in relation to age (Supplementary Tables 3, 4). Subgroups analyses of children and adults/adolescent between Fulanis and non-Fulanis showed significant differences in measures of alpha and beta diversity in the subgroups (Supplementary Tables 5, 6 and Supplementary Figures 5–7) except for alpha diversity in children (Supplementary Tables 5, 6 and Supplementary Figures 5, 6).

**TABLE 1:** Taxa with significantly different abundance on phylum, family, and genus level in the fecal microbiota between Fulani and Jarawa (non-Fulani) subjects (Wilcoxon rank test *p*-values were adjusted for multiple testing by FDR).

**Phylum**		**Fulani (mean% ± *SD*)**	**Jarawa (non-Fulani) (mean% ± *SD*)**	**FDR-corrected *p*-value**
	Bacteroidetes	**23.23 ± 0.14**	13.8 ± 0.09	0.04
	Spirochaetes	**0.43 ± 0.01**	0.10 ± 0.004	0.04
	Fusobacteria	**0.14 ± 0.006**	0.00 ± 0.00	0.046
	Actinobacteria	0.54 ± 0.005	**1.27 ± 0.016**	0.04
	Euryarchaeota	0.04 ± 0.001	**0.2 ± 0.002**	0.016
	Firmicutes	66.38 ± 0.18	** 77.63 ± 0.12**	0.047
	Lentisphaerae	0.00 ± 0.00	**0.09 ± 0.001**	0.0042
	Tenericutes	0.09 ± 0.0015	**0.36 ± 0.0034**	0.0098
	Verrucomicrobia	0.02 ± 0.0006	**0.20 ± 0.0045**	0.021
**Family**				
	Prevotellaceae	** 22.83 ± 0.14**	12.76 ± 9.62	0.013
	Bacteroidaceae	0.20 ± 0.005	**0.60 ± 0.014**	0.013
	Ruminococcaceae	13.11 ± 0.064	**20.09 ± 0.099**	0.044
	Christensenellaceae	0.18 ± 0.002	**1.18 ± 0.014**	0.012
**Genus**				
	*Eubacterium rectale* group	**2.72 ± 0.02**	1.37 ± 0.01	0.03
	*Campylobacter*	**0.13 ± 0.003**	0.01 ± 0.0004	0.035
	*Prevotella* 2	**2.85 ± 0.026**	0.96 ± 0.01	0.039
	*Bacteroides*	0.2 ± 0.005	**0.6 ± 0.014**	0.013
	*Ruminococcus* 1	0.41 ± 0.005	**1.11 ± 0.008**	0.011
	*Blautia*	2.96 ± 0.023	**6.64 ± 0.079**	0.045
	*Christensenellaceae* R7 group	0.18 ± 0.002	**1.17 ± 0.014**	0.025
	*Lachnospiraceae* ND 3007	0.04 ± 0.0005	**0.18 ± 0.0021**	0.036
	*Methanosphaera*	0.02 ± 0.0005	**0.09 ± 0.0013**	0.03

*Values in bold represent the group with enriched microbial taxa.*

### Predicted Functional Metagenomics Analysis

**FIGURE 5:**
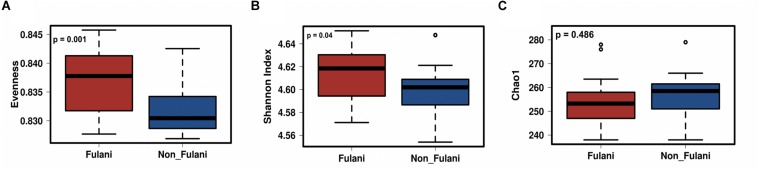
Differences in parameters of predicted functional alpha-diversity between Fulani and Jarawa (non-Fulani). Metagenomic predictions with PICRUSt showed a significantly higher predicted functional evenness index **(A)**, a significantly higher predicted functional diversity **(B)**, and no differences in the Chao1 index of predicted functional diversity **(C)** in the fecal microbiota of Fulani (red boxes) compared to Jarawa (non-Fulani, blue boxes). Mann–Whitney *U*-test was used with FDR-corrected *p*-values of 0.001 **(A)**, 0.04 **(B)**, and 0.486 **(C)**, respectively.

Metagenomic predictions using PICRUSt showed distinct microbial functional profiles in the Fulani and Jarawa (Table 2 and Supplementary Figure 3). Pathways involving lipopolysaccharide biosynthesis, ubiquinone and other terpenoid quinone synthesis, glycosyltransferases, arachidonic acid metabolism, riboflavin and vitamin B6 metabolism, and protein digestion and absorption were overrepresented in the Fulani fecal samples. In the Jarawa, the pathways involving methane metabolism; fructose and mannose metabolism; arginine and proline metabolism; pyruvate metabolism; and valine, leucine, and isoleucine biosynthesis were overrepresented. The predicted functional evenness (*p* = 0.001) and Shannon diversity (*p* = 0.04) of the gut microbiome was significantly higher in the Fulani than the Jarawa (Figures 5A,B), although the predicted functional alpha diversity of the Fulani gut microbiota was not significantly different from the Jarawa with Chao1 index (Figure 5C; *p* = 0.486).

**TABLE 2:** Top 30 discriminative metabolic pathways between the Fulani and Jarawa (non-Fulani) using PICRUSt (Wilcoxon sum rank test, *p*-values were adjusted for multiple testing by FDR).

**Metabolic pathway**	**FDR-corrected *p*-value**
**Pathways significantly enriched in the Fulani**	
Glutathione metabolism	0.044
*Vibrio cholerae* pathogenic cycle	0.015
Cell motility and secretion	0.029
Peroxisome	0.029
Isoquinoline alkaloid biosynthesis	0.046
D-Glutamine and D-glutamate metabolism	0.02
DNA replication proteins	0.044
**Pathways significantly enriched in the Jarawa (non-Fulani)**	
**Chloroalkane and chloroalkene degradation**	**0.0077**
**Methane metabolism**	**0.013**
**Nitrotoluene degradation**	**0.015**
**Xylene degradation**	0.023
Linoleic acid metabolism	0.023
**C5 branched dibasic acid metabolism**	**0.044**
Carbohydrate metabolism	0.044
**Fructose and mannose metabolism**	**0.025**
**Pentose phosphate pathway**	**0.036**
**Pyruvate metabolism**	**0.031**
**Dioxin degradation**	0.044
**Tetracycline biosynthesis**	**0.025**
**Sporulation**	**0.031**
Signal transduction mechanisms	0.029
Bisphenol degradation	0.043
**Germination**	**0.015**

*Bolded pathways were also significantly different in LEfSe analysis.*

### Predicted Organism-Level Microbiome Phenotypes Comparison

**FIGURE 6:**
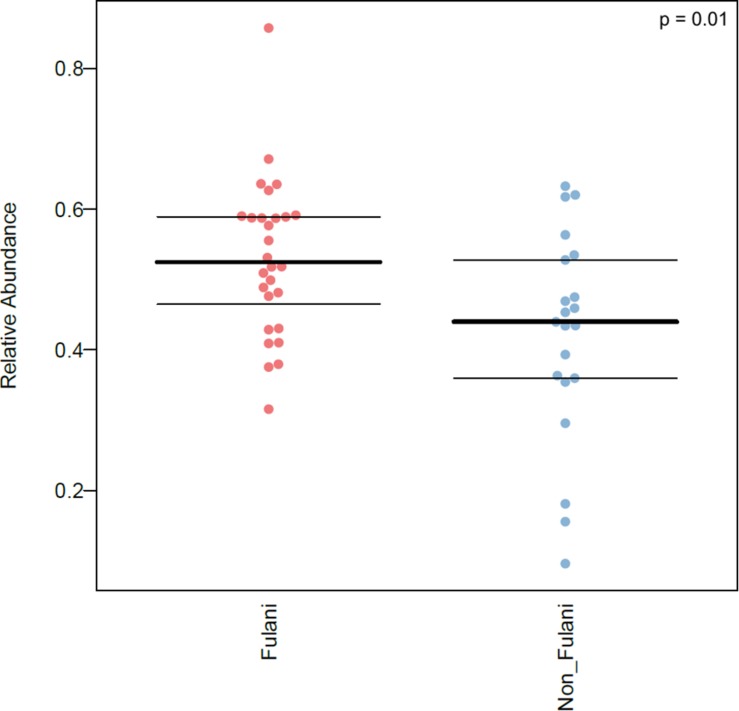
Relative abundance of microorganisms predicted to possess pathogenic potential in the intestinal microbiota of the Fulani (red) and the Jarawa (non-Fulani, blue) as determined by BugBase. Each dot represents the relative abundance of microorganisms with pathogenic potential in one individual sample, lines indicate mean ± *SD* (FDR-corrected *p*-value = 0.01164532). Mann–Whitney *U*-test was the statistical test used.

Predicted microbiome phenotypes using BugBase (Ward et al., 2017) revealed that the Fulani had a significantly higher abundance of microorganisms with predicted pathogenic potential (*p* = 0.01) (Figure 6). Bugbase is an algorithm that uses output from PICRUSt analysis (categorize_by_function.py) of 16S rRNA sequence data to predict biologically interpretable microbiome phenotypes, including the predicted relative abundance of microorganisms with pathogenic potentials. Also, the predicted relative abundance of oxidative stress-tolerant microbes in the Fulani tended to be higher than the Jarawa, but the difference did not reach statistical significance. The predicted high abundance of signatures of potentially pathogenic bacteria in the Fulani using BugBase also reflects PICRUSt results, which indicate the overrepresentation of pathways involved in the *Vibrio cholerae* pathogenic cycle (Table 2).

## Discussion

We investigated the gut microbiota of an African ethnicity, the Fulani, living a very unique traditional pastoral nomadic lifestyle. To study the potential effects of their lifestyle on the intestinal microbiota, we compared their fecal samples to a control group from an urban African population in the same area with a more westernized way of life. Apart from the recent study on rural Bassa and urbanized populations in Nigeria (Ayeni et al., 2018), and seven rural populations in Southern Cameroon (Morton et al., 2015), previous studies on the gut microbiome of Africans focused on the unique differences in the gut microbiome composition between Africans and industrialized populations. Examples of such studies include those of hunter–gatherer populations, traditional settled farming, or fishing populations (Schnorr et al., 2014; Gomez et al., 2016; Gupta et al., 2017). The lifestyle of the Fulani is somewhere between the first two of the aforementioned traditional populations as they have a nomadic lifestyle and their diet includes foods gathered in the wild as well as produced by traditional farming.

In general, we observed large differences in the taxonomic and functional composition in the intestinal microbiota between the Fulani and the urban Jarawa population, supporting previous observations that diet and lifestyle are major factors influencing the composition of the gut microbiota. Although we found some similarities to previous observations in traditional non-westernized populations, some findings were contradictory and unique in supporting a very specific lifestyle of the Fulani population.

The Fulani fecal microbiota had significantly less OTU richness and compositional diversity than the Jarawa. This is in contrast to previous comparative studies in other non-westernized populations in Africa, Asia, and South America (Schnorr et al., 2014; Clemente et al., 2015; De Filippo et al., 2017; Gupta et al., 2017; Nakayama et al., 2017). It is generally assumed that microbial diversity is reduced with westernization of the host community and that the higher diversity leads to a greater stability of the microbial ecosystem and better adaption to changes in the diet and exposure to parasites (Gupta et al., 2017). This interesting observation of lower alpha-diversity in the Fulani could be as a result of their narrow diet, unlike the Jarawa, who consume fibrous and fermented foods in addition to processed foods. Our report is in contrast to a recent study comparing the rural Bassa individuals and their urban counterparts in Nigeria, which reported, besides slightly more observed species in the rural population, no significant difference in the alpha-diversity of the gut microbiota between the study groups (Ayeni et al., 2018). Although microbial taxonomic diversity was significantly lower in the Fulani, their predicted functional diversity was significantly higher (albeit with Shannon index, not Chao1 index). A possible explanation for this observation is the acquisition of additional genes by horizontal gene transfer due to a high exposure to environmental microorganisms including pathogens that do not permanently colonize the human gastrointestinal tract. The result for predictive functional diversity is in agreement with a previous study demonstrating higher functional diversity by metagenomic analysis of uncontacted Amerindians compared with US persons (Yatsunenko et al., 2012).

Regarding taxonomic composition, the microbiota of the Fulani was enriched among others in Bacteroidetes, Spirochaetes, Fusobacteria, and Prevotellaceae and depleted in Firmicutes, Ruminococcaceae, Christensenellaceae, and *Blautia* (Table 1), similar to the findings of Ayeni et al. (2018) on rural Bassa individuals in Northern Nigeria. The significantly higher signatures of Bacteroidetes observed in the nomadic Fulani have also been reported in traditional societies practicing a hunter–gatherer lifestyle, while the observed enrichment of members of the Firmicutes in the Jarawa have been reported in societies that practice an agricultural- or industrial-based lifestyle (Gomez et al., 2016).

Although the less abundant Spirochaetes phylum was significantly enriched in the Fulani, there was no significant difference in the relative abundance of *Treponema* signatures. In contrast, previous studies on African populations have reported the high relative abundance of *Treponema* signatures in the gut of members of traditional societies, while this genus is significantly underrepresented in westernized societies (Schnorr et al., 2014; Gomez et al., 2016). Signatures of methanogenic members of Euryarchaeota have been established to be enriched in the gut of traditional African and semi-nomadic Amerindian societies (Walker, 2007; Clemente et al., 2015). However, we observed that Euryarchaeota members were significantly underrepresented in the Fulani, while they were enriched in the urban Jarawa study group. The methanogens have been reported to positively influence energy harvest by removing excess hydrogen that could inhibit ATP production (Walker, 2007). Since the Fulani consume fermented raw and cooked milk directly sourced from their herds, a higher relative abundance of *Lactobacilli* in the intestinal microbiota of the Fulani than the non-Fulani was assumed before the study; however, this was not the case. It could be that the access to and consumption of locally made yoghurt (nono) and *Lactobacillus* processed yoghurt by the Jarawa had also increased the relative abundance of *Lactobacilli* in their intestinal microbiota.

Overrepresentation in the Fulani of predicted pathways involved in lipopolysaccharide biosynthesis, ubiquinone and other terpenoid quinone synthesis, glycosyltransferases, arachidonic acid metabolism, riboflavin, and vitamin B6 metabolism was also in agreement with the study of the uncontacted Amerindians (Clemente et al., 2015). The presence of predicted pathways involved in vitamin metabolism could be related to the habitual consumption of fruits by the Fulani. In the Jarawa, overrepresentation of pathways involved in methane metabolism corroborates microbial taxonomic results, which indicate the significantly high abundance of archaeal signatures. The enrichment of KEGG pathways involved in the metabolism of simple sugar (fructose and mannose metabolism) and pathways involved in the metabolism of carbohydrates (such as the pentose phosphate pathway and pyruvate metabolism), which were overrepresented in the Jarawa, might be due to their consumption of more sugars and short-chain carbohydrates. An increase of these pathways has been shown to be associated with a western diet (Turnbaugh et al., 2009; Gomez et al., 2016). Although the *V. cholerae* pathogenic pathway was predicted to be enriched in the Fulani, *V. cholerae* was not identified in the microbial community by 16S RNA analysis. It is possible that certain resident microorganisms have acquired virulence genes involved in the pathogenic pathway from *V. cholerae* prior to its disappearance from the community to increase the fitness of the microbial recipients. The acquisition of virulence genes through horizontal transfer has been reported to contribute to increased fitness of naïve bacteria (Brown et al., 2006). Members of the genus *Prevotella* and *Campylobacter* (higher abundance in the Fulani) might have also been predicted to be potentially pathogenic by BugBase. Although *Prevotella* is associated with plant-based diet and is linked to beneficial gut health, it has been associated with certain extraintestinal diseases, especially those of the female reproductive tract (Ravel et al., 2011; Ma et al., 2012; Vieira et al., 2017; Freitas et al., 2018). Our functional data need to be interpreted with caution as they were generated using the prediction tool PICRUSt, which cannot replace metagenomic, metatranscriptomic, or metabolomic studies.

Our study has some limitations. First, the two groups studied showed a difference in age; in particular we had a higher proportion of children below the age of 11 years in the Fulani group than in the Jarawa group (Supplementary Table 2). Previous studies demonstrated that age is a factor that influences the composition of the intestinal microbiome and differences between children and adults have been reported (Koenig et al., 2011; Yatsunenko et al., 2012). It is generally assumed that at an age of 2–3 years, the intestinal microbiome of infants transforms into an adult-like microbiome (Koenig et al., 2011; Yatsunenko et al., 2012), probably by the change from breast feeding to a regular diet. In our study only two subjects (one in the Fulani and one in the Jarawa group) were below the critical age of 3 years; furthermore, analyses of our data for variation between adult versus pediatric microbiota showed no differences for alpha and beta diversity (Supplementary Tables 3, 4). In addition, when comparing subgroups of children and adults/adolescent between Fulanis and non-Fulanis we observed significant differences in measures of alpha and beta diversity in the subgroups (Supplementary Tables 5, 6 and Supplementary Figures 5–7) except for alpha diversity in children. The latter observation can be explained by the small samples size in this subgroup. It is therefore unlikely that dissimilarities in age between the two groups had a major impact on the differences observed in the intestinal microbiota between Fulani and Jarawa.

A second limitation is that we assessed antibiotic exposure only for a period of 1 month in the individuals included in the study. Although changes in alpha diversity after antibiotic intake normalize in healthy volunteers after 1 month (Burdet et al., 2019), effects of antibiotic intake on intestinal microbiota composition are even seen after 12 and more months (Rashid et al., 2015; Haak et al., 2019). It is possible that potential non-assessed differences in previous antibiotic intake between the two groups might have influenced the study results. It should be mentioned that this potential bias is also probably relevant for most studies investigating the intestinal microbiota, as self-reporting of previous antibiotic intake by patients is unreliable. In a previous study patients only correctly reported 50% of antibiotic courses in the last 6 months in response to an open question (Metlay et al., 2003).

Other confounding factors associated to changes in the microbiota such as BMI could also possibly explain the observed differences in the fecal microbiota between the two groups but were not assessed in the study.

Further functional metagenomic studies on these populations will be needed to support PICRUSt analysis predictions to achieve deeper insights into the functional capacity of the microbial community. Nevertheless, our study revealed insights into the impact of dietary practices and lifestyle on the composition of the gut microbiota of nomadic agro-pastoralists. It also revealed that a significantly higher taxonomic diversity of the microbial community is not always present in subjects living a non-Western traditional lifestyle and does not necessarily always translate to a significantly higher functional diversity.

## Materials and Methods

### Dietary Profile and Lifestyle of the Fulani and Jarawa

Verbal interviews with the Fulani community selected for the study revealed that they consume a diet rich in fiber such as grains (corn, millet, sorghum) (Supplementary Table 1), fruits from trees deliberately planted and those gathered from the wild, tubers, and other farm products, which is similar to previously reported dietary practices of rural Fulani in Nigeria (Lockett et al., 2009). In addition, they also regularly consume fermented drinks such as nono (fermented milk) and kunu (fermented millet), while they hardly eat meat outside of festival periods. Although they use salt, processed food additives are absent in the meals of the nomadic Fulani. They prefer to use additives derived from their crops (for example, additives from soy beans) to add flavor to their meals. The majority of the Jarawa are agriculturalists (subsistence farmers) and traders, and consume farm produce rich in fiber. However, their dietary habits are gradually being influenced by urbanization, and it is not uncommon for the Jarawa to include processed foods such as pasta, spaghetti, and fast foods in their daily diet. Also, to improve the aroma and the taste of their meals, artificial food additives are added during food preparation, mirroring the dietary practices of urban populations. The Jarawa consume foods of animal origin, such as fish, meat, and eggs, on a daily basis. Since the cultures of both Jarawa and Fulani are interwoven, some foods are commonly eaten by both groups such as local grains (*tamba*, *tuwo* varieties, and other grains) and fermented milk (known as nono or nunu) (Obi and Ikenebomeh, 2007; Okonkwo, 2011). Both groups also speak the Hausa language, which besides English is the language commonly spoken in Northern Nigeria. Nevertheless, both groups have their unique dialects. Historically, the nomadic Fulani and their herds have often been neglected by the local authorities with regard to the provision of health-care services, and this increases their vulnerability to zoonotic and non-zoonotic diseases (Sheik-Mohamed and Velema, 1999; Akogun et al., 2012; Muhammad et al., 2016). Unlike the Fulani, the Jarawa have easy access to health care, and the community studied lived about 1 km from the nearest hospital. Unlike the Fulani, the Jarawa have access to safer/potable drinking water, due in part, to proximity to the hospital. With the proximity of the Jarawa to a health-care center, they, unlike the nomadic Fulani, have easy access to health services and medications, including antibiotics (Sheik-Mohamed and Velema, 1999; Akogun et al., 2012; Muhammad et al., 2016).

### Subject Enrollment

Participants included 28 healthy Fulani volunteers (2–70 years) from a community of Fulani living in Pabaman-shanu village, Plateau State, North-Central Nigeria. Since the participants were illiterate, verbal consent was obtained by the participants, and this was documented by a separate witness. Twenty-two (22) consenting healthy Jarawa (non-Fulani) participants (2–65 years) living in the same geographical area (Lamingo, Jos, Plateau State) were also recruited for this study. General (sample, age, sex, group) information on enrolled subjects can be found in Supplementary Table 2. Based on age, the volunteers in each of the study groups were sorted into two groups: children (≤11 years) and adolescent and adults (>11 years). Based on this classification, 17 adolescents and adults and 5 children comprised the population of the non-Fulani volunteers, while 15 adolescents and adults and 13 children comprised the population of the Fulani volunteers. Written informed consent was obtained from the subjects and the parents of children and adolescents enrolled.

The volunteers were healthy and had not received antibiotics for at least 1 month before sampling. Because subjects were uneducated, we chose to assess the composition of their diet by verbal interviews rather than questionnaires.

### Sampling Procedure

Fecal samples were collected from the study participants, transferred into 50 ml tubes, and preserved with 95% ethanol. After 24–36 h, the ethanol in each tube was carefully decanted and the bolus was transferred aseptically into another sterile DNA-free container half-filled with 3 mm-sized silica gel beads and sterile cotton wool (on the top of the beads, for desiccation) according to previously described protocols (Nsubuga et al., 2004; Schnorr et al., 2014). The whole process was done at room temperature. The samples were transported by courier to the Centre for Medical Research (ZMF), Medical University of Graz, Austria where they were stored at −80°C until further processing.

### DNA Isolation, 16S rRNA Gene Amplification and Sequencing

Total genomic DNA was isolated from previously frozen samples using a combination of mechanical and enzymatic lysis followed by bacterial DNA extraction with the MagnaPure LC DNA Isolation Kit III (Bacteria, Fungi; Roche, Mannheim, Germany) according to previously described methods (Klymiuk et al., 2016; Kump et al., 2018) on a MagNA Pure LC 2.0 instrument (Roche Diagnostics, Mannheim, Germany). Hypervariable region V4-specific PCR amplification was performed as described by Klymiuk et al. (2016) using the primer set 515F-5′-GTGCCAGCMGCCGCGGTAA and 806R-5′-GGACTACHVGGGTWTCTAAT synthesized at Eurofins (MWG, Ebersberg, Germany). PCR reaction for each sample was performed in triplicates. Amplicons were purified as previously described (Klymiuk et al., 2016), quantified, pooled, and finally sequenced on a MiSeqII desktop sequencer (Illumina, Eindhoven, Netherlands) using V3 chemistry (MiSeq Reagent Kits v6, Illumina, Eindhoven, Netherlands) and a paired-end protocol according to manufacturer’s instructions.

### Microbiota Analysis and Statistics

The raw reads (in FASTQ format obtained from next-generation sequencing) were processed using QIIME (version 1.9.1, if not specified otherwise) on the internal Galaxy software (version 1.24.0) provided by the ZMF at the Medical University of Graz (Caporaso et al., 2010; Afgan et al., 2016). Briefly, paired-end reads were first merged for further processing. Joint raw sequences were filtered by removing sequence reads shorter than 200 bps, reads containing ambiguous bases, or reads with an average quality score of <29. In addition adapters were identified and removed with Cutadapt (v1.16) (Martin, 2011). Pre-filtered sequences were then screened for potential chimeras by USEARCH v6.1 (Edgar, 2010) against the 97% clustered SILVA reference 16S rRNA gene database (v128) (Pruesse et al., 2007; Quast et al., 2012). Remaining high quality sequence reads were finally processed by the QIIME open reference pipeline (pick_open_reference_otus.py, default setting if not specified otherwise) to perform clustering into OTUs at 97% sequence similarity. SILVA (v128) was used as reference database for taxonomic classification, and UCLUST for subsequent *de novo* OTU clustering strategies. Singletons were rejected prior to final downstream analysis. Taxonomic composition, alpha- [observed species, Chao1 index, Shannon index, phylogenetic diversity (PD) whole tree, Simpson index] and beta diversity (Bray–Curtis, unweighted and weighted UniFrac) analysis were assessed using the core diversity workflow (core_diversity.py) of QIIME. Diversity analysis, here in particular Evenness calculation, was performed using Calypso which implements Evenness using vegan (R package, Oksanen et al., 2019) as Pielou’s measure of species evenness (*J*) with J = *H*/log(*S*); *S* = species richness and *H* is the Shannon Weiner diversity. For diversity analysis data were rarified to a minimal depth of 13,100 sequences per sample. Further downstream analysis, visualizations, and statistical calculations, such as RDA (OTU level) and boxplots, and Adonis or ANOSIM, were performed using Calypso (v7.38) (Zakrzewski et al., 2017). Also, the impact of age on the gut microbiota of both populations was tested with the aforementioned methods. Phylogenetic Investigation of Communities by Reconstruction of Unobserved States (PICRUSt version 1.0.0) was applied to predict metagenomic content from the 16S rRNA gene data (Langille et al., 2013; Kanehisa et al., 2016). RDA plots were constructed for beta diversity using Bray–Curtis distances, and functional diversity was measured using the Shannon index on PICRUSt-predicted metagenomes. Finally, BugBase was used to assess the predicted phenotypes present in microbiome samples (Ward et al., 2017). As this software estimates the proportion of Gram-positive, Gram-negative, potentially pathogenic, biofilm-forming, and oxidative stress-tolerant microbial signatures, it provides interesting insights into the pathogenic potential of the investigated communities. Differentially abundant feature testing was implemented by QIIME within the core_diversity workflow (group_significance.py) and applying Kruskal–Wallis. For this analysis false discovery rate (FDR) correction was used to adjust *p*-values for multiple comparisons. Statistics on alpha-diversity metrices was assessed by QIIME within the core_diversity workflow (compare_alpha_diversity.py) by applying a non-parametric pairwise *t*-test (*p*-values are calculated using 999 Monte-Carlo simulations). Here Bonferroni correction was applied to adjust *p*-values for multiple comparisons. Significant differences in microbial composition between Fulani and non-Fulani beta diversity were investigated by multivariate analysis using ANOSIM/Adonis implemented and provided by Calypso based on the distances (Bray–Curtis, unweighted UniFrac) calculated by QIIME. Significance between Firmicutes/Bacteroides ratio was determined using Pearson’s Chi-squared test with simulated *p*-value (based on 1*e* + 06 replicates). In addition, Calypso redundancy analysis was used to explore the association between community composition and multiply explanatory variables (group and age). Taxonomic and functional discriminatory features as well as differences in pathogenic capacity, obtained from BugBase, between the Fulani and non-Fulani were determined using LEfSe.

LDA score (log10) > 2 (LEfSe) and *p*-values < 0.05 (general) were considered statistically significant. Default settings were used, if not otherwise specified.

Age distribution between Fulani (19.4 ± 16.14 mean/*SD*) and non-Fulani (29 ± 18.03 mean/*SD*) ranges from 2 to 70 years in both groups, see Supplementary Table 2. Difference in age between the groups was tested using the Wilcoxon–Mann–Whitney-test, resulting in a *p*-value = 0.0398. Based on this statistically significant difference, age was critically investigated with regard to alpha and beta diversity for young (age ≤ 11) and adult (age > 11) Fulani and non-Fulani. None of these calculations resulted in a statistically significant result, see Supplementary Tables 3, 4.

## Ethics Statement

This study was carried out in accordance with the recommendations of the National Code for Health Research Ethics, University of Ibadan/University College Hospital Ethics Committee with written informed consent from all subjects. All subjects gave written informed consent in accordance with the Declaration of Helsinki. The study protocol was approved by the ethical review committee of the University of Ibadan/University College Hospital on September 9, 2016 with NHREC/05/01/2008a, and reference number: UI/EC/16/0211.

## Author Contributions

AA was involved in formal analysis, investigation, methodology, visualization, and writing of the original draft. FA was involved with conceptualization, formal analysis, investigation, methodology, project administration, and editing of the draft. CH was involved with formal analysis, investigation, methodology, visualization, project administration, and editing of the draft. BH was involved in formal analysis, methodology, visualization, and review/editing of the draft. CM and GG were involved with investigation, methodology, and review/editing of the draft.

## Conflict of Interest Statement

The authors declare that the research was conducted in the absence of any commercial or financial relationships that could be construed as a potential conflict of interest.
