# Supplementary material for: Impact of a Nomadic Pastoral Lifestyle on the Gut Microbiome in the Fulani Living in Nigeria
Source: Front Microbiol. 2019 Sep 13;10:2138. doi: 10.3389/fmicb.2019.02138 (PMC6753190; doi:10.3389/fmicb.2019.02138)

Supplementary Table 1. Overview of general lifestyle and food ingredients used by Fulani and the Jarawa (Non-Fulani) derived from verbal interviews, additional details on the differences in diet are provided in the material and methods section.

	**Fulani**	**Jarawa (Non-Fulani)**
**Food**		
Breakfast	Beverage:<br>Kunu (a fermented drink made of mixed ground grains) or Local herbs, Dowrori (Milk), Biradan (raw fermented milk), Kosan (cooked fermented milk)<br>Main dish:<br>Any solid dish made from ground grains such as maize, white and yellow millet along with karkachi (a local vegetable soup)	Tamba (ground local grains), processed foods (meat pie, cake, white bread)
Lunch	*Gote* (a local porridge made of ground maize and spinach) is the most preferred option. Other dishes include yam, local rice, cabbage	Parboiled/refined rice, beans, or grain dishes (*Gaari Masara*, *Dawa*), Cassava (*Gaari rogo*), yam (*Bawan doya*)
Dinner	*Tuwo shinkafa* (a solid dish made with rice grain), or other *tuwo* (made from other grains) together with bitter leaf soup	Two dishes, or pasta (indomie, spaghetti)
**Lifestyle**		
General	Nomads, rural environment	Living in a city
Profession	Agriculturalists and traders	Agriculturalists, civil servants, traders, and students
Access to medical supply	They do not have easy access to medical supply. They have to travel to Jengre, a nearby town to gain access to medical supply	Close to a tertiary hospital (Jos University Teaching Hospital, Jos, Plateau State, Nigeria)
Water supply	Water obtained from stream for	Water obtained from the

	domestic activities<br><br>Water obtained from the borehole built by a non-governmental organization (NGO) in 2016 (a few months before the commencement of the study)	borehole and well.

Supplementary Table 2. Summary of metadata, including age, for enrolled subjects.

Sample	Group	Age [years]	Sex	Sample	Group	Age [years]	Sex
A01	Non-Fulani	11	Female	A24	Fulani	25	Male
A02	Non-Fulani	15	Male	A25	Fulani	Adult*	Male
A03	Non-Fulani	30	Male	A26	Fulani	11	Female
A04	Non-Fulani	23	Female	A27	Fulani	Adult*	Male
A06	Non-Fulani	28	Female	A28	Fulani	9	Female
A07	Non-Fulani	11	Female	A29	Fulani	6	Male
A08	Non-Fulani	20	Male	A30	Fulani	70	Female
A09	Non-Fulani	27	Male	A31	Fulani	7	Female
A10	Non-Fulani	2	Female	A32	Fulani	10	Female
A11	Non-Fulani	23	Female	A33	Fulani	15	Female
A12	Non-Fulani	5	Female	A34	Fulani	9	Female
A13	Non-Fulani	35	Male	A35	Fulani	4	Female
A14	Non-Fulani	53	Female	A36	Fulani	25	Female
A15	Non-Fulani	56	Male	A37	Fulani	6	Female
A16	Non-Fulani	37	Female	A38	Fulani	2	Male
A17	Non-Fulani	56	Female	A39	Fulani	10	Male
A18	Non-Fulani	65	Female	A40	Fulani	26	Female
A19	Non-Fulani	48	Female	A41	Fulani	8	Male
A20	Non-Fulani	27	Male	A42	Fulani	34	Male
A21	Non-Fulani	9	Female	A43	Fulani	7	Male
A22	Non-Fulani	28	Male	A44	Fulani	25	Female
A23	Non-Fulani	Adult*	Male	A45	Fulani	12	Male
				A46	Fulani	12	Female
				A47	Fulani	32	Female
				A48	Fulani	8	Male
				A49	Fulani	40	Male
				A50	Fulani	45	Male
				A51	Fulani	37	Female

* Subjects were adults, the information on their exact age was not provided by these individuals

Supplementary Table 3. Summary of p-values [adjusted p-values] for alpha diversity differences between children (age <= 11) and adolescent/adult (age > 11) in the Fulani and the Non-Fulani group. Statistical significance was assessed using a two-sided non-parametric t-test at a rarefication depth of 13100. Benjamini-Hochberg procedure was applied to correct for multiple testing comparisons.

	**Fulani** *(children vs. adolescent/adult)* p-value [p-value-adjusted]		**Non-Fulani** *(children vs. adolescent/adult)* p-value [p-value-adjusted]	
Chao1	0.5788	[0.5788]	0.3980	[0.4451]
Observed_species	0.5114	[0.5788]	0.3539	[0.4451]
PD_whole_tree	0.3107	[0.5788]	0.4451	[0.4451]
Shannon	0.4793	[0.5788]	0.2080	[0.4160]
Simpson	0.3358	[0.5788]	0.2080	[0.4160]
Simpson_reciprocal	0.3358	[0.5788]	0.2080	[0.4160]

Supplementary Table 4. Summary of p-values [adjusted p-values] for beta diversity differences between children (age <=11) and adolescents/adults (age > 11) in the Fulani and the Non-Fulani groups. Statistical significance was assessed by ANOSIM. Benjamini-Hochberg procedure was applied to correct for multiple testing comparisons.

	**Fulani** *(children vs adolescent/adult)* p-value [p-value-adjusted]		**Non-Fulani** *(children vs adolescent/adult)* p-value [p-value-adjusted]	
Bray-Curtis	0.29	[0.435]	0.6	[0.63]
Unweighted UniFrac	0.71	[0.71]	0.63	[0.63]
Weighted Unifrac	0.071	[0.213]	0.329	[0.63]

Supplementary Table 5. Summary of p-values [adjusted p-values] for alpha diversity differences between children (age <= 11) and adolescent/adult (age > 11) in the Fulani and the Non-Fulani group. Statistical significance was assessed using a two-sided non-parametric t-test at a rarefication depth of 13,100. Benjamini-Hochberg procedure was applied to correct for multiple testing comparisons.

	**Children Fulani vs. children Non-Fulani** p-value [p-value-adjusted]		**Adolescent/adult Fulani vs adolescent/adult Non-Fulani** p-value [p-value-adjusted]	
Chao1	0.38	[0.665]	0.056	[0.056]
Observed_species	0.5	[0.7]	0.017	[0.0198]
PD_whole_tree	0.118	[0.665]	0.003	[0.00525]
Shannon	0.31	[0.665]	0.0015	[0.0035]
Simpson	0.73	[0.79]	0.0063	[0.008]
Simpson_reciprocal	0.79	[0.79]	0.00054	[0.00189]
Evenness	0.36	[0.665]	0.00097	[0.000679]

Supplementary Table 6. Summary of p-values [adjusted p-values] for beta diversity differences between children (age <=11) and adolescents/adults (age > 11) between the Fulani and the Non-Fulani. Statistical significance was assessed by ANOSIM. Benjamini-Hochberg procedure was applied to correct for multiple testing comparisons.

	**Children Fulani vs. children Non-Fulani** p-value [p-value-adjusted]		**Adolescent/adult Fulani vs adolescent/adult Non-Fulani** p-value [p-value-adjusted]	
Bray-Curtis	0.007	[0.021]	0.012	[0.012]
Unweighted UniFrac	0.018	[0.027]	0.007	[0.0105]
Weighted Unifrac	0.057	[0.057]	0.005	[0.0105]

Supplementary Table 7: Summary of differential taxa enriched in the Fulani and Non-Fulani from LEfSe analysis (p-values were determined with Wilcoxon's Rank Test).

Phylum		Fulani (Mean % ± SD)	Non-Fulani (Mean % ± SD)	p-value	LDA Score
	Bacteroidetes	23.23 ± 14	13.8 ± 9	0.04	5.0
	Firmicutes	66.38 ± 18	77.63 ± 12	0.047	5.0
	Actinobacteria	0.54 ± 0.5	1.27 ± 1.6	0.04	3.5
Class					
	Clostridia	50.62 ± 15.63	65.05 ± 17.44	0.0055	5.0
	Bacteroidia	22.09 ± 13.81	13.81 ± 9.26	0.021	5.0
Order					
	Clostridiales	50.62 ± 15.63	65.05 ± 17.44	0.0055	5.0
	Bacteroidales	22.09 ± 13.81	13.81 ± 9.26	0.021	5.0
Family					
	Lachnospiraceae	20 ± 8.63	29.3 ± 15. 55	0.019	4.75
	Ruminococcaceae	13.11 ± 6.40	20.09 ± 9.90	0.044	4.5
	Christensenellaceae	0.18 ± 0.2	1.18 ± 1.4	0.012	4.0
Genus					
	Blautia	2.96 ± 2.3	6.64 ± 7.9	0.045	4.25
	Ruminococcaceae UCG002	0.4 ± 0.52	1.7 ± 1.74	0.0012	4.10
	Christensenellaceae R7 group	0.18 ± 0.2	1.17 ± 1.4	0.025	4.0
	Eubacterium coprostanoligenes group	0.5 ± 0.38	1.4 ± 1.46	0.019	3.7
	Ruminococcus 1	0.4 ± 0.5	1.1 ± 0.81	0.0031	3.6
	Veillonella	1.7 ± 2.84	0.6 ± 1.24	0.028	3.8
	Eubacterium rectale group	2.72 ± 2	1.37 ± 1	0.03	3.9
	Alloprevotella	2.2 ± 2.06	0.9 ± 1.16	0.0046	4.0
	Prevotella 2	2.85 ± 2.6	0.96 ± 1	0.039	4.1
	Prevotella 9	15.9 ± 9.72	10.2 ± 7.57	0.035	4.7

Supplemental Figure 1: Relative Abundance of Microbial Taxa in the Gut of Fulani and Non-Fulani at the Phylum level

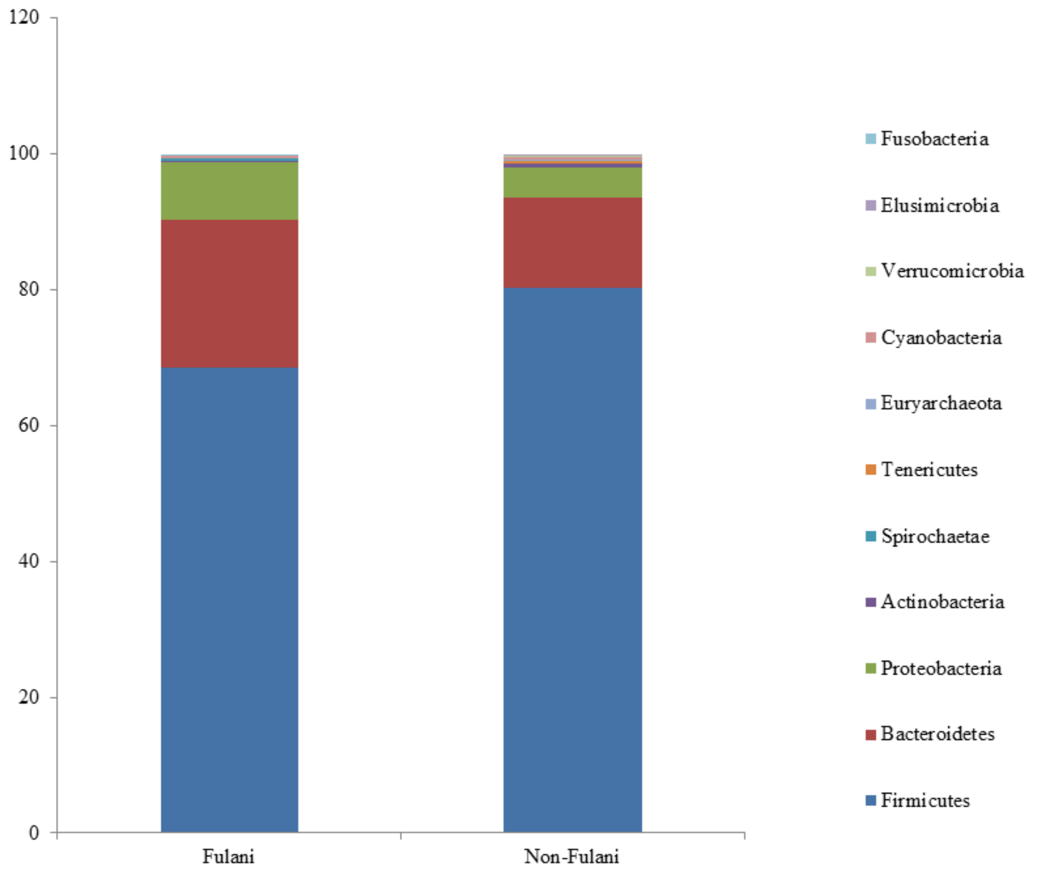

Supplemental Figure 2. Differences in abundance of microbial taxa in the fecal microbiota of Fulani (red bars) and Non-Fulani (blue bars). Bars that exceed the LDA score of 2 depict significant different abundance of the particular microbial taxon in the fecal microbiota between the two study groups.

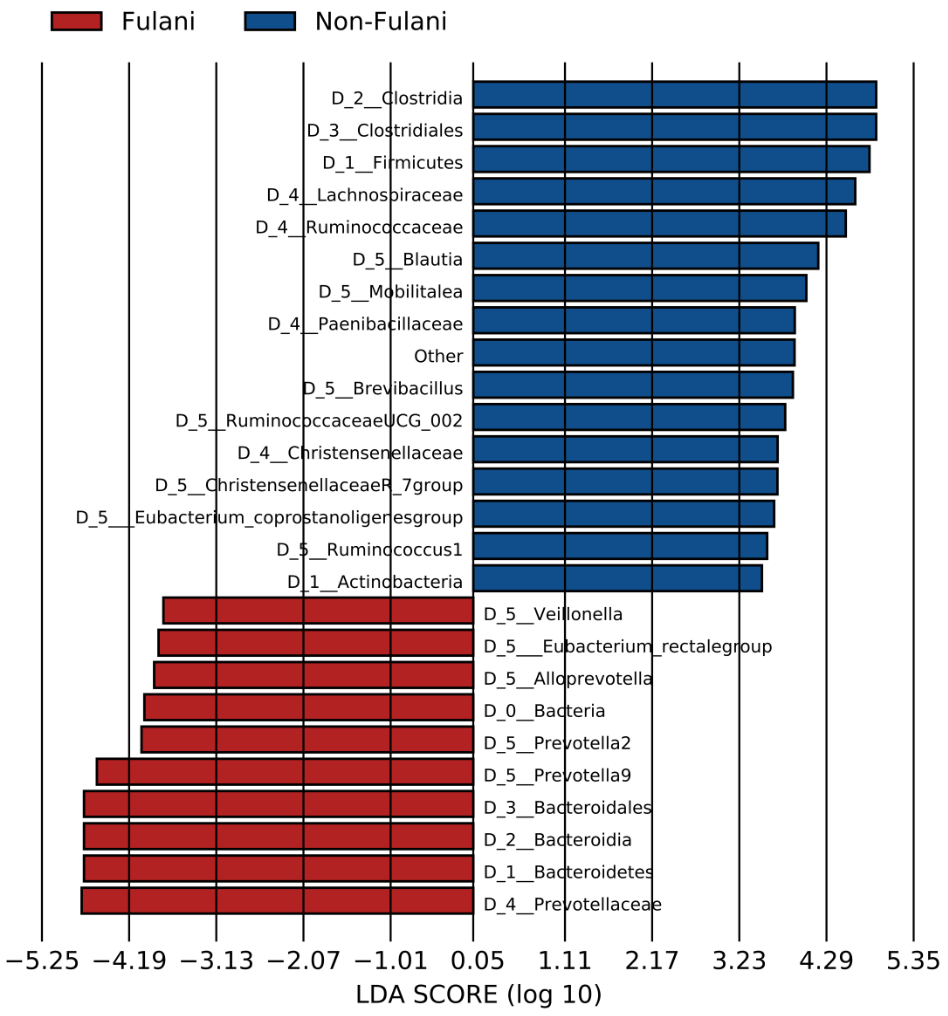

Supplemental Figure3. Predictive enrichment of significantly different functional capabilities of the microbiome of Fulani (red) and Non-Fulani (blue). Bars that exceed the LDA score of 2 depict significant abundance of the particular predicted pathway in the fecal microbiota between the two study groups.

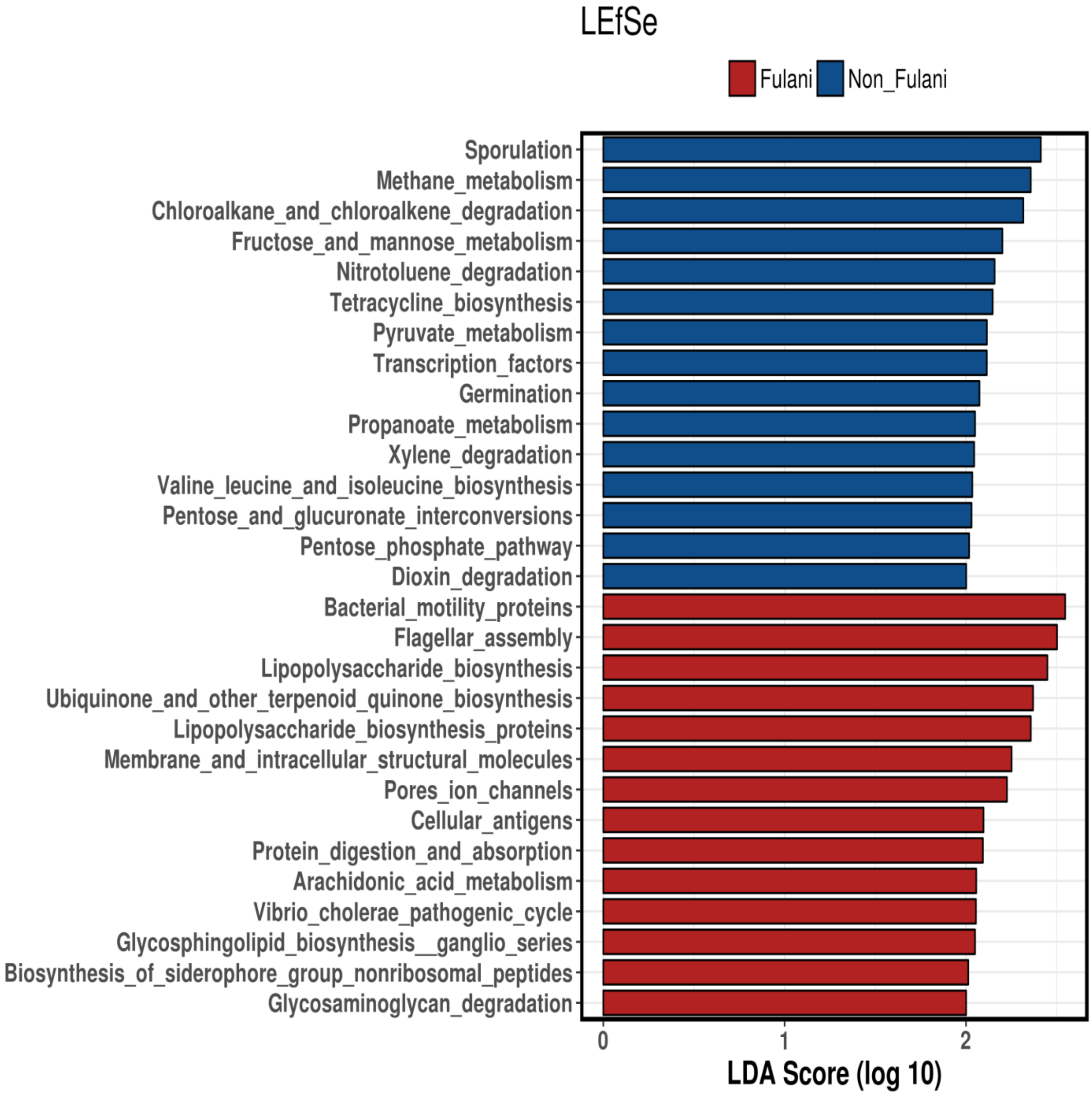

Supplemental Figure 4. RDA plot based on Bray-Curtis distances indicating children ≤ 11 years in both groups

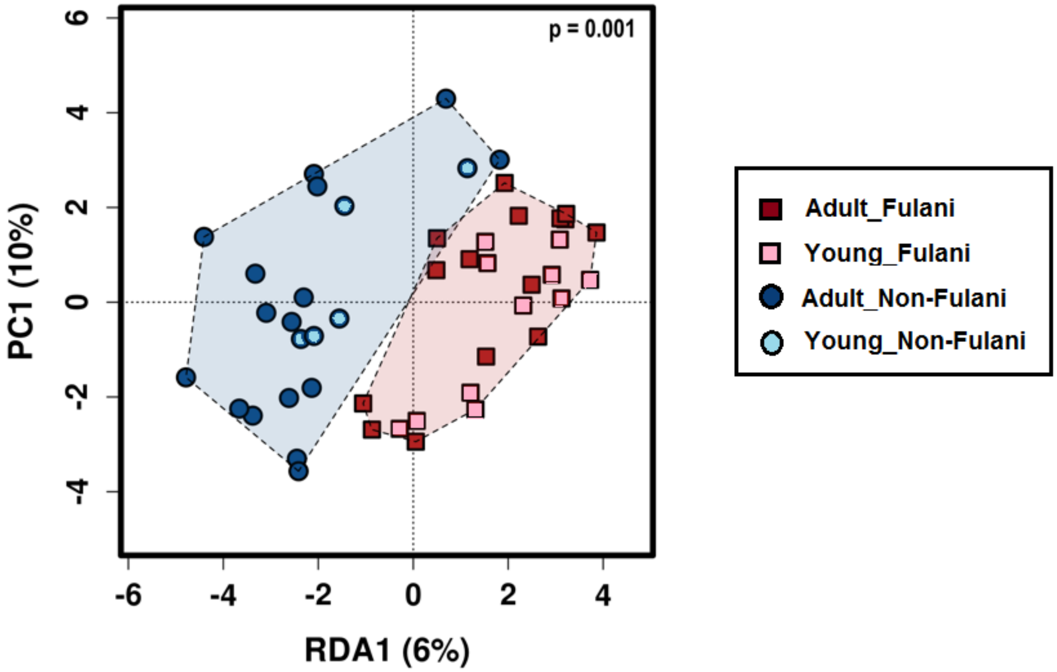

Supplemental Figure 5. Beta diversity. RDA plot based on Bray-Curtis distances shows patterns of significant separation between the Fulani (red boxes) and the Jarawa (Non-Fulani, blue circles) children at the OUT level (p = 0.005). ANOSIM of Bray curtis distances for the Fulani and non-Fulani children: R = 0.337, p = 0.019

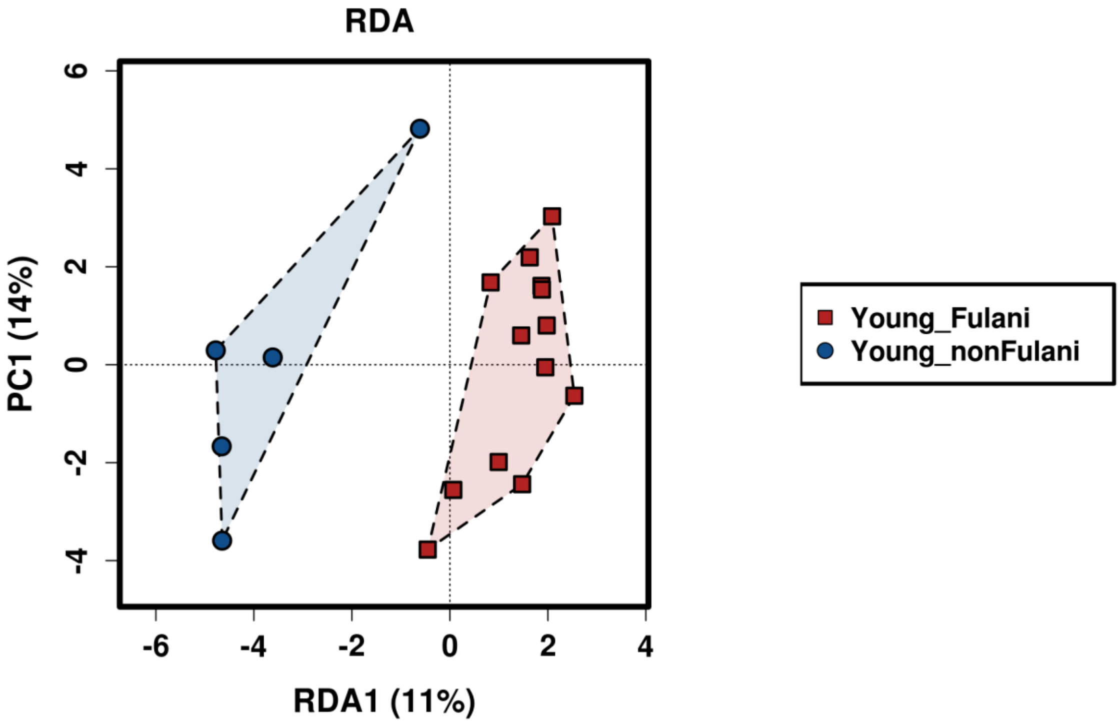

Supplemental Figure 6. . Beta diversity. RDA plot based on Bray-Curtis distances shows patterns of significant separation between the Fulani (red boxes) and the Jarawa (Non-Fulani, blue circles) adolescents/adults at the OUT (p = 0.001) ANOSIM of Bray Curtis Distances for the Fulani and non-Fulani adults: R = 0.123, p = 0.004

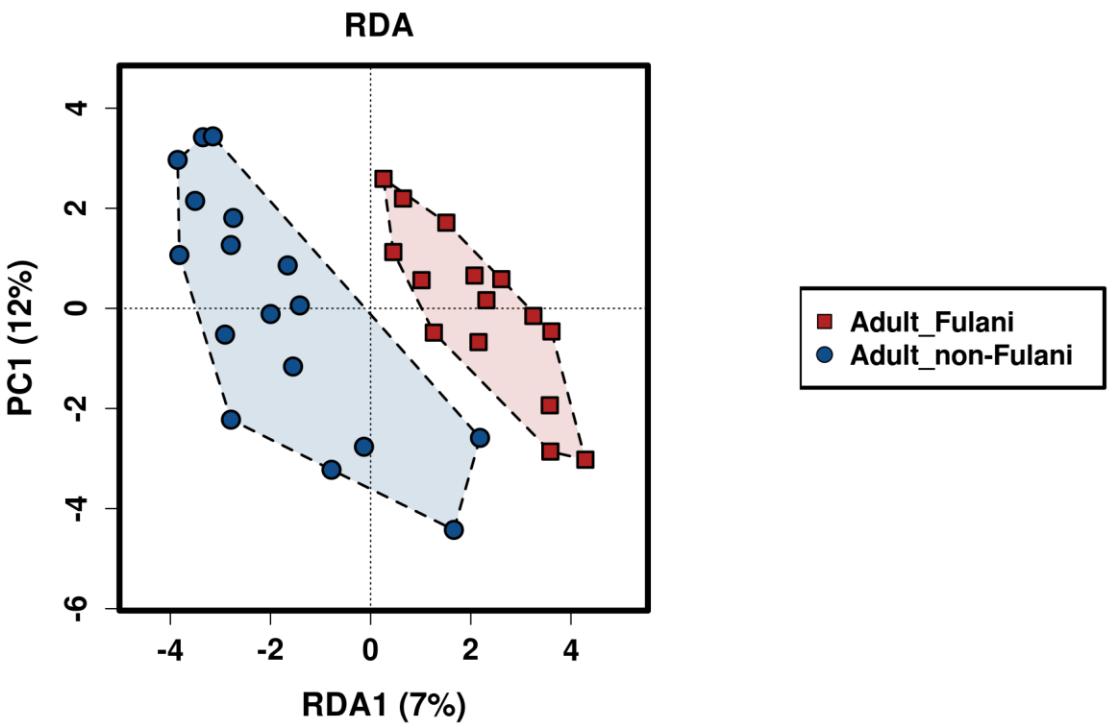

Supplemental Figure 7. Alpha diversity across age. Red dots indicate Fulani, blue dots non Fulani.

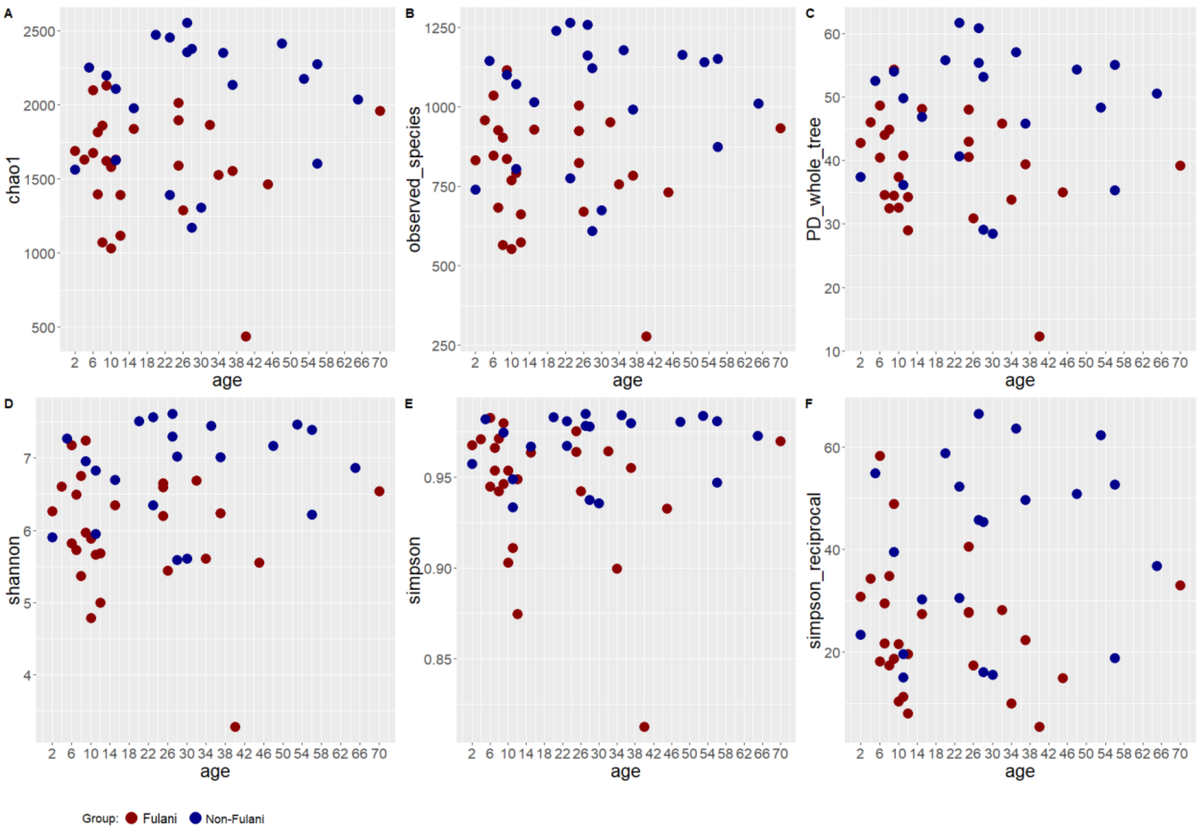

Supplement: Supplementary file 1 [file Data_Sheet_1.PDF]
